# Peritonitis Following Examination for Uterine Polypus

**Published:** 1845-10

**Authors:** 


*Peritonitis following examination for Uterine Polypus.*—At a recent meeting of the Société de Chirurgie, M. Lenoir presented the uterus of a woman, 55 years of age, who had been suddenly seized with peritonitis, the day after an examination, by which the presence of an uterine polypus in the vagina had been ascertained. She had been labouring under hemorrhage for seven or eight months. The existence of the polypus was ascertained without the slightest difficulty. M. Lenoir intended to have operated on the following day, but was prevented by the development of peritonitis, which carried off the patient within eight days.

M. Malgaigne narrated a similar case. He was called to Versailles to see a lady who was affected with uterine polypus. He practised the *toucher*, and recognised, without the slightest difficulty, the presence of a polypus, which he intended to have extirpated. The following day, however, she was seized with peritonitis and died.—*Gazette des Hôpitaux*, as quoted in *Lancet* of 26th July, 1845.

[When the parts are morbidly sensitive, examination by the toucher is always attended with more or less risk, and should, if possible, be delayed till this state has been removed, by rest and soothing treatment. Want of attention to this caution, a little accidental roughness in performing the toucher, or idiosyncrasy, are the causes of the occurrence of such cases as the above, which, though rare, teach a useful lesson.]—*London and Edinburgh Monthly Jour.*

